# Case report: CD7-targeted autologous CAR-T therapy for the treatment of T-cell acute lymphoblastic leukemia undergoing allogeneic peripheral blood stem cell transplantation in the long-term follow-up

**DOI:** 10.3389/fimmu.2024.1469251

**Published:** 2024-11-15

**Authors:** Lian-Lian Li, Yue-Ping Xia, Qianqian Li, Peng Wang, Ping-Ping Sun, Xing-Guang Wang, Rui Zhang

**Affiliations:** ^1^ Department of Hematology, Cangzhou People’s Hospital, Cangzhou, China; ^2^ Department of Clinical Lab, Cangzhou People’s Hospital, Cangzhou, China; ^3^ Department of Pathology, Cangzhou People’s Hospital, Cangzhou, China; ^4^ Department of Hematology, Qilu Hospital Of Shandong University Dezhou Hospital, Dezhou, China

**Keywords:** CD7 CAR-T cells, T-cell acute lymphoblastic leukemia, allogeneic peripheral blood stem cell transplantation, relapse, measurable residual disease

## Abstract

**Introduction:**

Chimeric antigen receptor T-cell (CAR-T) therapy has emerged as a promising approach for treating relapsed/refractory (r/r) T-cell acute lymphoblastic leukemia (T-ALL). However, it is mostly used as a bridging therapy for allogeneic hematopoietic stem cell transplantation (allo-HSCT). Furthermore, secondary allo-HSCT is costly and associated with significantly high treatment-related mortality rate than the primary transplants. In this report, we present the case of an adult T-ALL patient who underwent CD7-targeted autologous CAR-T cell therapy that was not bridged with secondary allo-HSCT.

**Case presentation:**

The adult T-ALL patient relapsed for a third time after undergoing allogeneic peripheral blood stem cell transplantation (PBSCI) and achieving the first complete remission (CR1). Therefore, the patient was administered CD7-targeted autologous CAR-T cell therapy that was not bridged with secondary allo-HSCT. Towards this, the endogenous CD7-deleted CAR (CD7-CAR7) T cells were generated using CRISPR/Cas9 gene-editing technology. However, after the first infusion, the CD7 CAR-T cells did not show significant proliferation when analyzed at two weeks and the patient became positive for peripheral measurable residual disease (MRD). Therefore, after a two-week period, an augmented dose of CAR-T cells was administered. MRD was monitored in the peripheral blood and bone marrow samples. The patient achieved complete remission and did not require targeted treatment after the completion of CD7 CAR-T-cell therapy. The current follow-up data has shown that the patient is negative for MRD and has been disease-free for more than 42 months.

**Conclusion:**

The results of this case study provide evidence for the long-term efficacy of CD7-targeted autologous CAR-T-cell therapy without requiring secondary allo-HSCT in patients with r/r T-ALL that have relapsed after previous allogeneic PBSCT.

## 1 Introduction

Acute lymphoblastic leukemia (ALL) is characterized by the malignant growth of immature T/B cells and is the most common malignancy among children. T-cell ALL (T-ALL) is an aggressive cancer developing from the progenitor T-cells. Among ALL patients, the incidence rate of T-ALL is 10-15% in children and 25% in adults (1, 2). Malignant relapse and death are reported for approximately 30% of T-ALL patients within 2 years of diagnosis. At present, relapsed T-ALL patients are primarily treated with high-intensity chemotherapy and cancer immunotherapeutic agents. The new epigenetic agent chidamide has been approved by the US Food and Drug Administration for the treatment of relapsed/refractory (r/r) T-ALL, but complete remission (CR) is reported in only 20-30% of the treated patients (3–5). Moreover, patients receiving single-agent therapy have a median survival of only 8 months. Therefore, there is an urgent need to discover novel and innovative therapeutic strategies for T-ALL.

Chimeric antigen receptor-expressing T cells (CAR-T cells) have emerged as an effective type of targeted tumor immunotherapy with high targeting efficacy, durability, and tumor destruction ability in both *in vitro* experiments and clinical trials. CAR-T-cell immunotherapy has shown significant therapeutic efficacy in treating B-cell hematological malignancies and is considered superior to other immunotherapies under investigation (6–8). In recent years, CAR-T-cell immunotherapy has also shown significant efficacy in the treatment of T-cell hematological malignancies. CD7 is a transmembrane glycoprotein belonging to the immunoglobulin superfamily. CD7 expression is higher in the malignant T cells than in the normal T cells from healthy donors. Therefore, CD7 is considered as an attractive target for CAR T-cell therapy (9). However, the use of chimeric antigen receptor (CAR) T cell therapy targeting CD7 as a novel immunotherapy for the treatment of (r/r) T-ALL faces major challenges and is in the early stages (10, 11).CAR-T therapy is a transitional treatment for patients with r/r ALL, but the therapeutic effects last only for 3 months to 1 year (12). Previously, donor-derived CD7-directed CAR-T cells were used as a bridging treatment for haploidentical stem cell transplantation and significantly improved the long-term disease-free survival rate of patients with r/r T-ALL (9, 10, 13). However, without the subsequent follow-up of allogeneic hematopoietic stem cell transplantation, the survival rate of the patients rarely was approximately 1 year. Allogeneic hematopoietic stem cell transplantation (HSCT) is highly risky with significant costs. Moreover, treatment-related mortality of a second allogeneic HSCT is often higher than the primary allogeneic HSCT.

In this report, we describe the case of an adult patient with T-ALL who relapsed after allogeneic peripheral blood stem cell transplantation (PBSCT). The patient was treated with autologous anti-CD7 CAR-T-cell infusion. After the first infusion, the patient relapsed multiple times. The patient was resistant to most drugs but achieved complete remission after second CD7-CAR-T treatment for over 3 years without requiring a second allogeneic hematopoietic stem cell transplant. This case study results suggested that some relapsed T-ALL patients may achieve long-term survival with CD-7-CAR-T treatment.

## 2 Case report

### 2.1 Medical history

A 17-year-old young male patient with headache was previously diagnosed with T-ALL at the Peking University First Hospital in 2018. Bone marrow smear analysis showed 94% blasts. Immunohistochemistry analysis demonstrated that the blasts were positive for CD34 (71.3%), CD38, CD3, CD7, CD2, CD5, CD8, cCD3, and cTDT. Cytogenetic analysis exhibited a normal male karyotype of 46XY. Next-generation sequencing analysis did not detect any fusion genes. The patient underwent four courses of chemotherapy before achieving the first complete remission (CR1). Subsequently, he received allogeneic peripheral blood stem cell transplant (PBSCT) from his 4/6 HLA-matched sister and did not show any apparent rejection after recovery. During the five-month treatment period, the patient did not show bone marrow relapse or measurable residual disease (MRD) positivity. Subsequently, the patient experienced the first relapse of ALL. He achieved the second CR (CR2) after receiving two courses of vindesine-daunorubicin-cyclophosphamide-pegylated enzyme-prednisone (VDCLP) chemotherapy, but experienced relapse again. He achieved a third CR (CR3) after two courses of VCDLP chemotherapy, but experienced a third relapse of ALL during the final course of consolidation chemotherapy (The detailed treatment timeline prior to CAR T cell therapy is provided in **Supplementary Material 1**).

### 2.2 Clinical findings and autologous CAR-T-cell therapy

**Figure 1:**
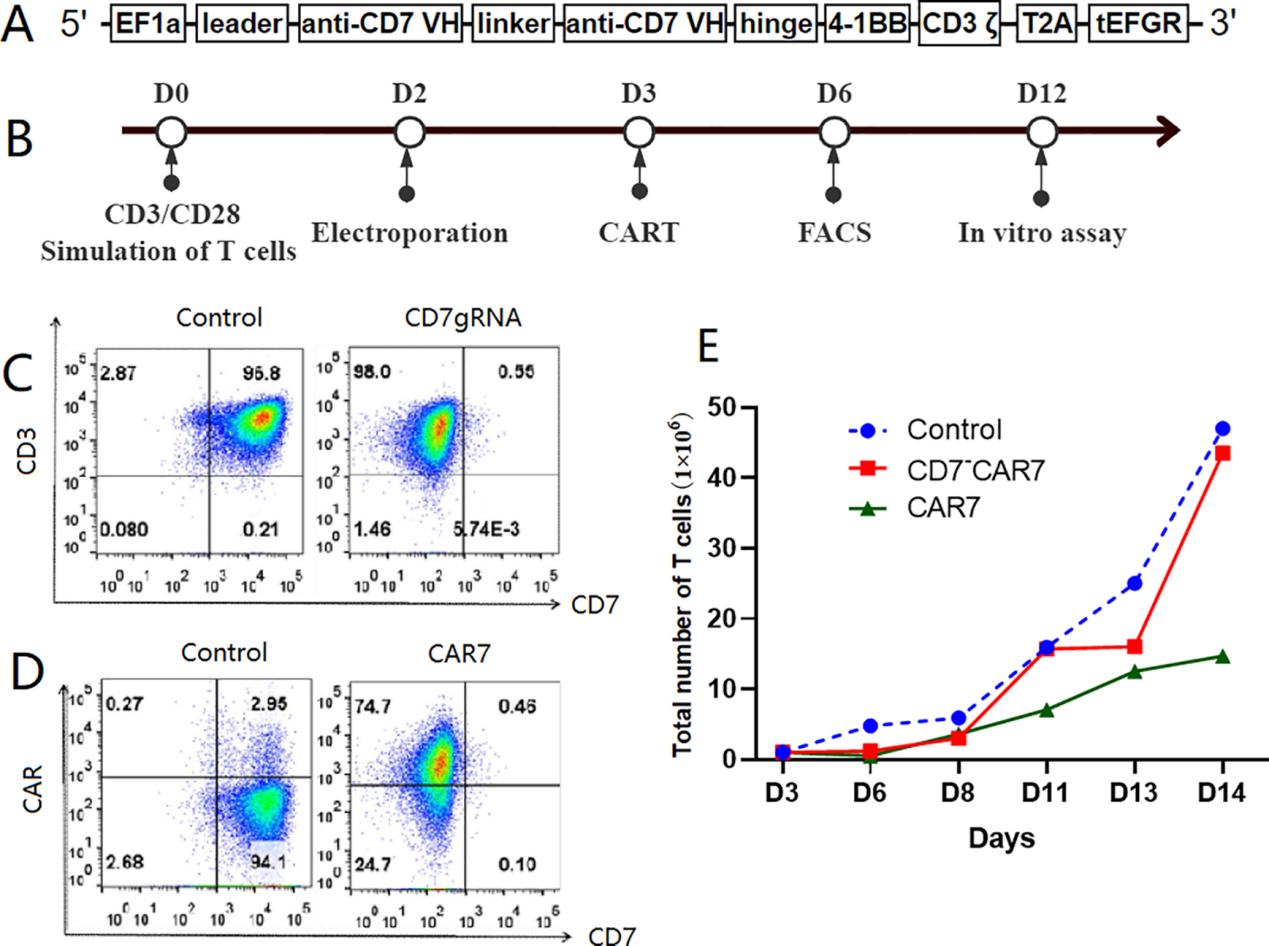
Preparation of CAR-T cells targeting CD7. **(A)** Diagrammatic representation of the *CD7* gene. CD7-deleted CAR (CD7^-^CAR7) T cells were generated by knocking down CD7 expression on the CDs of the CAR-T cells via CRISPR/Cas9 genome editing. In brief, a lentiviral CAR structure targeting CD7 was constructed. The anti-CD7 single chain variable fragment (scFv) fragment was ligated into the lentiviral vector in frame with the 4-1BB and CD3ζ domain sequences in a second-generation CAR. The main chain connected the extracellular tEGFR domain with T2A and was used to detect CAR expression in the T cells. **(B)** Flow chart shows the strategy for generating the gene-edited CD7-CAR-T cells. This includes preparation of endogenous CD7-deficient CAR (CD7~CAR7) T cells targeting CD7 and T-cell activation with CD3/CD28-conjugated magnetic beads for two days before removal. **(C)** Flow cytometry analysis of the knockout efficiency of CD7gRNA in the T cells. As shown, CD7 expression on the T cells decreased from 97% to 0.55% on the 6^th^ day, which was three days after electrotransfecting the Cas9 protein and CD7-gRNA complex along with the lentivirus encapsulating the CD7-CAR structure. **(D)** High expression of CD7-CAR on the T cells. **(E)** Amplification plot of the gene-edited CD7-CAR-T cells. As shown, there was a consistent expansion trend for the gene-edited CD7-CAR-T cells (CD7 CAR-T cells) in comparison with the control T cells. The low expansion rate of the CAR-T cells without endogenous CD7 knockdown was because of mutual killing. CAR, chimeric antigen receptor; FACS, fluorescence-activated cell sorting.

In October 2020, the patient was referred to our center and recruited into the clinical trial for CD7 CAR-T-cell therapy with a costimulatory 4-1BB endodomain (NCT number: NCT05619861; www.clinicaltrials.gov). The CD7-deleted CAR (CD7^-^ CAR7) T cells were obtained from the Hebei Senlang Biotechnology Co., Ltd. The flow chart of the protocol for generating the CD7 CAR-T cells using CRISPR/Cas9 genome editing according to a previous study (14) are shown in **Figure 1**. On the 14^th^ day, peripheral blood samples were collected for generating the engineered CAR-T cells. Before CAR-T cell therapy, bone marrow biopsy demonstrated strong CD7 positivity and aberrant myeloproliferative phenotype. About 79% of the nucleated cells were primitive and immature lymphocytes expressing significant levels of CD3, CD8, CD2, CD5, and CD7. Furthermore, 76% of the abnormal cells were nucleated.

### 2.3 Treatment regimen

**Figure 2:**
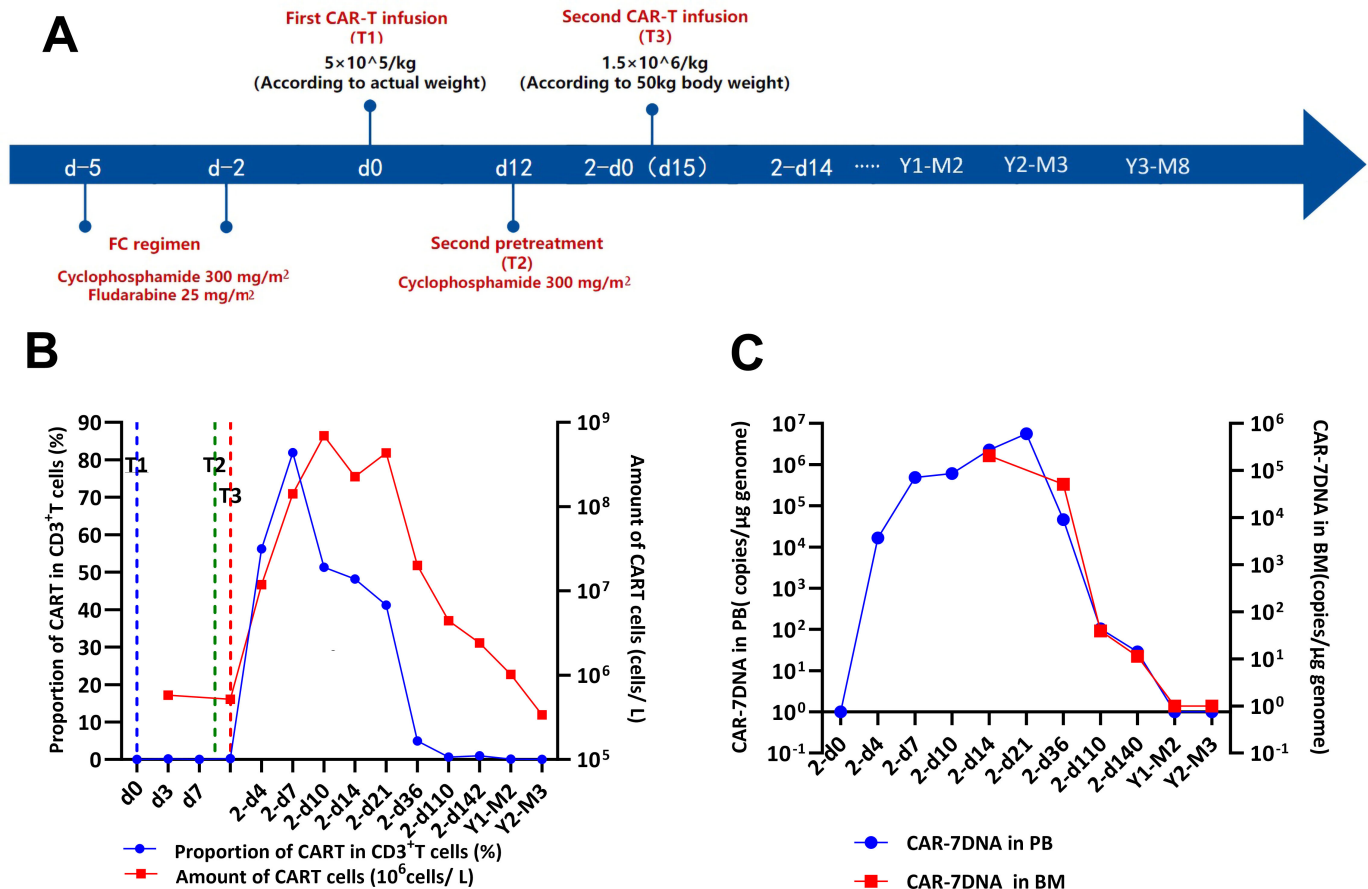
Efficacy of the CD7 CAR-T-cell therapeutic regimen. **(A)** Timeline of disease progression and relevant treatments. **(B)** Flow cytometry analysis of CD7 expression and the persistence of CD7 CAR-T cells during the treatment. **(C)** QRT-PCR analysis of CD7 expression and the persistence of CD7 CAR-T cells during the treatment. CAR-T cells, chimeric antigen receptor-modified T cells; d0, time of the first CAR-T-cell infusion; 2-d0, time of the second CAR-T-cell infusion; T1, first CAR-T-cell infusion; T2, second CAR-T-cell infusion; T3, third CAR-T-cell infusion.

Details of the treatment regimen and follow-up are shown in **Figure 2A**. The patient received lymphodepleting chemotherapy with fludarabine (25 mg/m^2^) and cyclophosphamide (300 mg/m^2^) for 3 days (days -4 to -2). Subsequently, the patient was infused with a single dose of CD7 CAR-T cells (5×10^5^ cells/kg) for 30 min on d0. Then, the patient received an intramuscular injection of 25 mg promethazine (defined as “T1”). The vital signs were closely monitored throughout the process and until 12 h after the injection.

### 2.4 Response to treatment protocol

**Figure 3:**
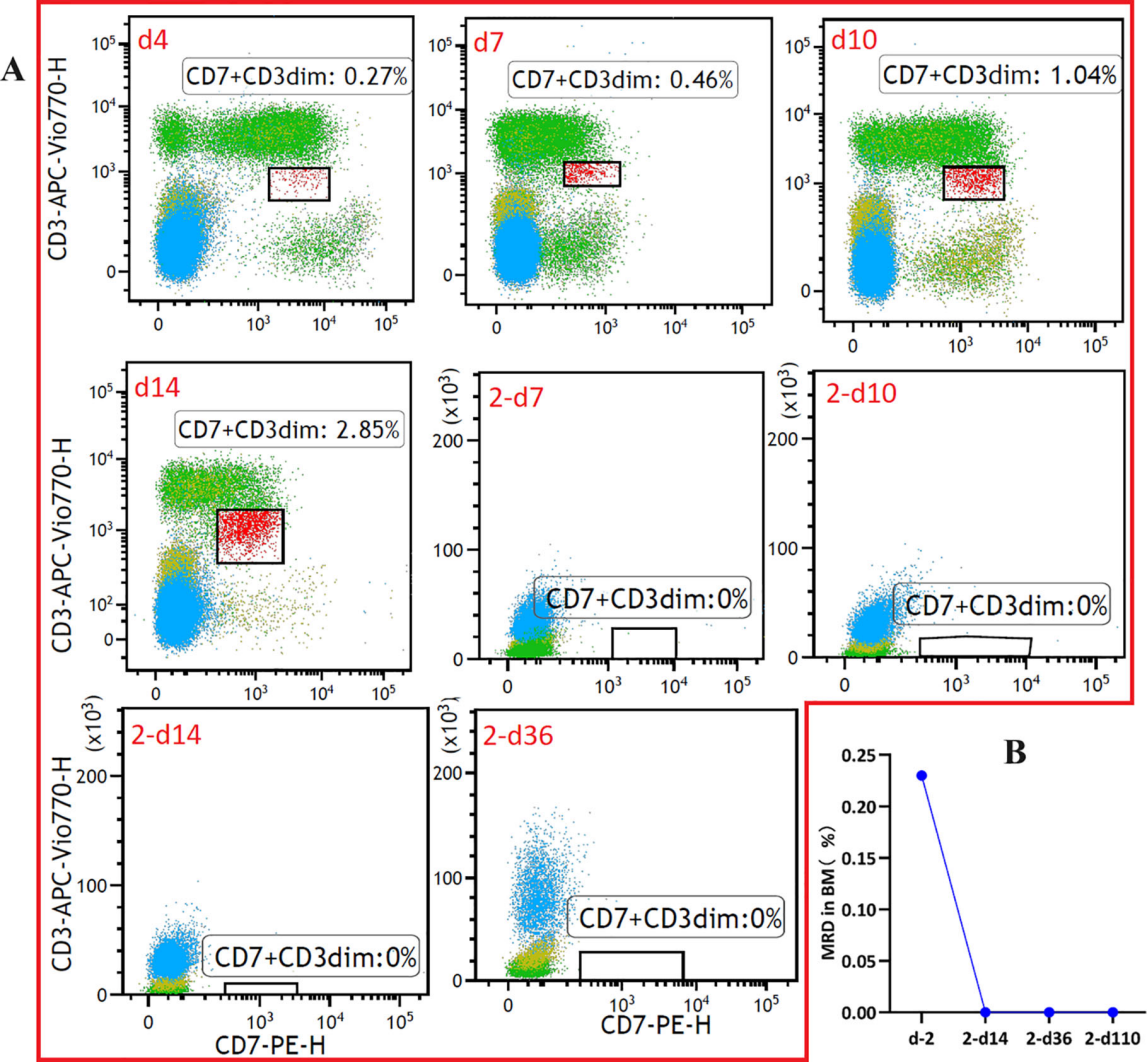
Monitoring of measurable residual disease (MRD) in the **(A)** peripheral blood and **(B)** bone marrow samples at different time points after CAR-T-cell infusion using flow cytometry (The flow cytometry data for T cells after CAR-T can be found in **Supplementary Figures S3**-**6**).

**Figure 4:**
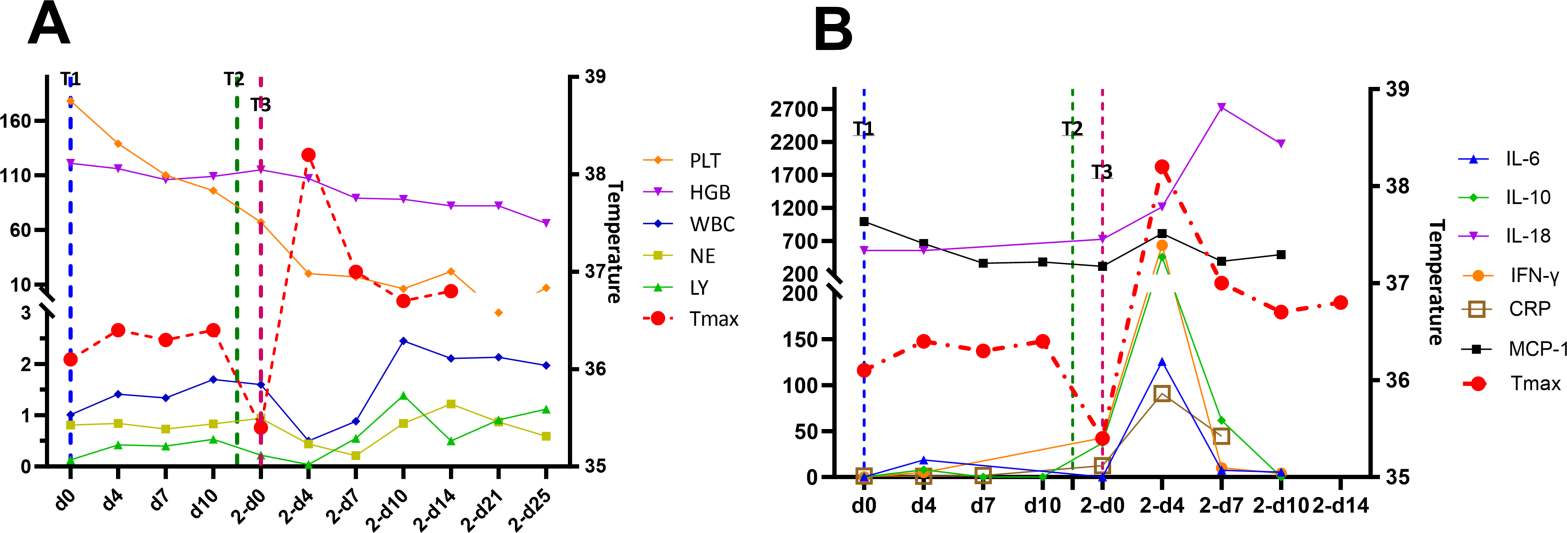
**(A)** Hemogram test results and temperature curves and **(B)** cytokine levels during the treatment regimen. The values in parentheses represent the normal range of each parameter. T1, first CAR-T-cell infusion; T2, second CAR-T-cell infusion; T3, third CAR-T-cell infusion.

The patient did not experience increased body temperature or fever after the CAR-T cell infusion process. Biochemical parameters returned to normal by day 14 after the CAR-T cell infusion and CAR-T-cell proliferation was detected in the peripheral blood through flow cytometry (**Figure 2B**) and qPCR (**Figure 2C**). Peripheral blood MRD was detected on days 4 (d4), 7 (d7), and 10 (d10) after CAR-T-cell infusion and tended to increase (**Figure 3A**). This was probably caused by insufficient number of CAR-T cells in the first infusion and their ineffective amplification in the patient. Therefore, the patient was administered another single dose (second dose) of 1.5×10^6^ CAR-T cell infusion. Subsequently, the patient was administered 300 mg/m^2^ cyclophosphamide for two consecutive days (days -4 to -2; defined as “T2”). To distinguish from the first infusion, the time after the second infusion was represented as “2 day+0 (2-d0, defined as “T3”). The patient developed febrile syndrome from 2-d2 to 2-d7. The peak body temperature was 38.4°C at 05:00 AM on 2-d4 and the blood pressure decreased to 90/50 mmHg at 07:00 AM on 2-d7. This indicated low-grade cytokine release syndrome (CRS). The hemogram test results demonstrated a gradual decrease in the platelet (PLT) counts and hemoglobin (HGB) levels in the peripheral blood, and other hematological indices exhibited minimal fluctuations (**Figure 4A**). Furthermore, the highest cytokine levels were concomitant with the peak body temperature on 2-d4, whereas IL-18 levels peaked on 2-d7 (**Figure 4B**). However, the patient did not show severe symptoms of CRS or tumor lysis syndrome. The patient’s fever abated after administration of anti-infective and infusion supportive therapy. His blood pressure also returned to normal. Furthermore, he did not develop CAR-T-cell-associated encephalopathy syndrome (**Supplementary Table S1**). The peripheral blood and bone marrow samples were negative for MRD on 2-d4 and 2-d14, respectively. Moreover, aberrant leukemia cells were not detected in the bone marrow and peripheral blood samples on 2-d36 and 2-d110 (**Figure 3**). Flow cytometry analysis showed 0.23% of aberrant cells in the bone marrow aspirates on the day before (d-1) CAR-T-cell therapy (**Figure 4**). The bone marrow smears and biopsies on 2d-4, 2d-36, and 2d-110 were negative for MRD and leukemia cells were not detected (**Figure 3B**).

During the monthly posttreatment check-ups in the last two months, there were no signs of leukemia relapse. However, it is possible that the cumulative toxicity of pretreatment chemotherapy may result in a longer recovery period for patients with pancytopenia after the second CAR-T-cell infusion. The patient was discharged on 2d-28 after recovering completely. According to the follow-up data, the patient has been disease-free for more than 42 months.

## 3 Discussion

CD7 is a transmembrane glycoprotein that is highly expressed in more than 95% of lymphoblastic leukemias and lymphomas, and peripheral T-cell lymphoma (PTCL) (15). CD7 is also expressed in the peripheral T cells, natural killer (NK) cells, and their precursors (16). When patients with T-cell malignancies were treated with immunotoxin-loaded monoclonal antibodies targeting CD7, severe CD7-related toxicity was not observed but the tumor response was relatively mild (17). In CD7-targeted CAR-T-cell therapy, incomplete downregulation of CD7 on the transduced CAR-T cells causes a phenomenon called “fratricide” wherein the CAR-T cells kill each other because of CD7 expression, thereby significantly reducing the proliferation of CD7 CAR-T-cells and lowering their therapeutic efficacy (14, 18, 19). Since CD7 expression in the endogenous T cells can lead to mutual killing of the CD7 CAR-T cells, we used the CRISPR/Cas9 gene-editing technique to significantly reduce the expression of CD7 on the CAR-T cells and generate endogenous CD7-deleted (CD7-CD7-) T cells against the CD7-CAR (CD7-CAR7) T cells.

We report a rare case of CD7-targeted autologous CAR-T-cell therapy following relapse of T-ALL after allogeneic PBSCT. Recently, Zhang et al. (20) evaluated the outcomes of CD7 CAR-T-cell therapy in four patients with T lymphoblastic leukemia/lymphoma (T-ALL/LBL) and one patient with NK/T-cell. Among these, two patients had undergone at least 3 lines of intensive chemotherapy, one patient had experienced relapse after autologous stem cell transplantation, and another patient had relapsed after allogeneic hematopoietic stem cell transplantation (allo-HSCT). After CAR-T-cell therapy, 4 patients achieved CR and one patient recovered partially. Furthermore, one patient died because of abdominal infection after complete recovery. We observed comparable results using an innovative CD7 CAR-T-cell therapeutic strategy that was different from the one reported in the study by Zhang et al. In our study, the first CD-7 CAR-T-cell infusion did not significantly increase the CD-7 CAR-T-cell counts after two weeks and the peripheral blood MRD of the patient was positive and severe. Therefore, higher doses of CD-7 CAR-T cells were infused again after two weeks. The results of MRD monitoring using bone marrow and peripheral blood samples demonstrated that the patient achieved CR.

During CD-7 CAR-T-cell therapy, the patient did not demonstrate symptoms of a severe cytokine storm. After the second CD-7 CAR-T-cell infusion, the patient experienced intermittent fever and low blood pressure between 2-d4 and 2-d7. However, the adverse symptoms improved after anti-infection treatment. This may be caused by a significant reduction in tumor burden after receiving chemotherapy and before CD-7 CAR-T-cell infusion. This may also have been an effect of two CD-7 CAR-T-cell infusions. The patient should have developed immune tolerance after the first CAR-T-cell infusion. The toxicity caused by CAR-T-cell therapy is difficult to predict and control. As a result, most phase I clinical trials use a conservative dose-escalation strategy for CAR-T-cell infusion (21, 22).

The World Health Organization (WHO) has recommended that a CD7-targeting universal chimeric antigen receptor (UCAR) designed with third-generation signaling domains (CD28 and 4-1BB costimulatory structural domain) can be considered as a potential strategy for the treatment of r/r T-ALL and non-Hodgkin T-cell lymphoma. This strategy was first proposed by Cooper et al. (18). Human T cells are genetically edited using CRISPR/Cas9 technology to remove both CD7 and the T-cell receptor (TCR) α chain (TRAC) to generate universal CAR-T cells (CD7 UCAR-T cells), which target cancer cells expressing CD7 without attacking the healthy T cells. TRAC deficiency impairs TCR-mediated signaling and prevents suicidal T-cell death as well as the graft-versus-host disease (GVHD), which is life-threatening. In the absence of fungicides or T-cell-mediated GVHD, allogeneic CD7 UCAR-T cells exhibit robust anticancer activity against human T-ALL cell lines and primary T-ALL cells derived from patients in both *in vitro* and *in vivo* experiments. Although these findings need to be further investigated before clinical application, they provide important insights for the development of an effective T-cell-based pericyte therapy for malignant tumors without the need for autologous T cells.

The patient in our study had already experienced multiple relapses before being referred for treatment. The patient’s family declined the option of a second allogeneic transplant because of financial burden, lack of donors, and concerns about the risks associated with a second transplant.

This study demonstrated that complete remission of more than 3 years can be achieved after CD7-CAR-T treatment without undergoing a second allogeneic HSCT. This suggested that some T-ALL patients who relapse after CD-7-CAR-T treatment may achieve long-term survival.

Allogeneic hematopoietic stem cell transplantation is associated with significant risks and inflated costs. Moreover, compared to the first transplant (SCT1), second allogeneic hematopoietic stem cell transplantation (SCT2) is riskier and is typically associated with a higher treatment-related mortality (TRM) rate. A study reported that the 3-year non-relapse mortality (NRM) was significantly higher for SCT2 compared to SCT1 (40.4% vs.20%) (23, 24). However, a second transplant is unavoidable in some cases. Our data shows that patients that experience relapse after undergoing allogeneic hematopoietic stem cell transplantation may achieve remission with CD7-CAR-T therapy and may not require a second allogeneic hematopoietic stem cell transplant. Further investigations are required to investigate changes in the status of T-cell chimerism after CD7-CAR-T therapy and in-depth study of the underlying mechanisms with large cohort prospective data.

Furthermore, we aim to follow-up the patient further to comprehensively assess the clinical efficacy of CD7-CAR-T therapy by recording the remission rate and survival time of this patient. We also will further investigate the implications of CD7-CAR-T therapy by including more similar cases in the future, whenever available.

## Data Availability

The original contributions presented in the study are included in the article/**Supplementary Material**. Further inquiries can be directed to the corresponding author.
