# Supplementary material for: Case report: CD7-targeted autologous CAR-T therapy for the treatment of T-cell acute lymphoblastic leukemia undergoing allogeneic peripheral blood stem cell transplantation in the long-term follow-up
Source: Front Immunol. 2024 Nov 15;15:1469251. doi: 10.3389/fimmu.2024.1469251 (PMC11604605; doi:10.3389/fimmu.2024.1469251)
Supplement: Supplementary file 1 [file DataSheet1.docx]

1. **The detailed treatment timeline prior to CAR T cell therapy**

The patient, a male, initially presented with headache in July 2018 and was diagnosed with acute T-cell lymphoblastic leukemia at Peking University First Hospital, with central nervous system involvement and liver, spleen, and kidney involvement. On August 8, 2018, he achieved complete remission (CR) after VDCLP chemotherapy. Subsequently, he received consolidation therapy with VDCLP, high-dose MTX, and HyperCVAD A/B regimens. On January 11, 2019, he underwent conditioning with TBI+VP16+Bu/Cy+ATG. From January 23 to 25, 2019, he received a hematopoietic stem cell transplant from his sister (HLA 4/6 match), consisting of bone marrow and peripheral blood stem cells (total MNCs 9.7*10^8/kg, CD34+ cells 4.7*10^6/kg). White blood cell and platelet engraftment occurred as expected. During the transplant process, he experienced mucositis, intestinal Candida tropicalis infection, cytomegalovirus infection, and liver function abnormalities, which improved with symptomatic treatment. Post-transplant bone marrow examinations showed continued remission of the primary disease with complete donor chimerism. After intermittent intrathecal chemotherapy, tumor cells in the cerebrospinal fluid turned negative for central nervous system leukemia.

On 2019-06-20, bone marrow re-examination showed: extremely active bone marrow proliferation, with lymphocytes accounting for 99%, mostly primitive lymphoblasts, indicating relapse. Bone marrow flow cytometry results showed: R5 group accounted for 76.31% of all nucleated cells, expressing CD3, CD8, CD2, CD5, CD7, and partially expressing CD4 and CD34.

The patient then sought treatment at our hospital. On 2019-07-30, VDCP regimen was administered, followed by VDCLP, high-dose MTX, and VDCLP chemotherapy regimens. The evaluation showed complete remission (CR).

On 2020-08-20, bone marrow aspiration showed: active proliferation level 1, with immature lymphocytes accounting for 10% of nucleated cells. Bone marrow flow cytometry: abnormal T lymphocytes accounted for 11.05% of nucleated cells. The disease relapsed again. High-dose MTX was administered but with poor results.

On 2020-09-12: Bone marrow reexamination: Abnormal T lymphocytes account for approximately 70.60% of all nucleated cells (**Figure S1**)

On 2020-09-13: VDCLP regimen administered: Epirubicin 40mg d1-3 (based on patient's condition), Ifosfamide 1000mg d1-15, Pegaspargase 3750u d5-19, Dexamethasone 10mg d1-14, 5mg d15-21. On 2020-10-13, bone marrow aspiration showed: Grade I active proliferation of nucleated cells, increased proportion of erythroid cells. Flow cytometry of bone marrow showed: MRD: 0.23% (**Figure S2**). Pre-CAR-T cerebrospinal fluid (CSF) tumor cells were negative.

On 2020-10-16, FC regimen administered: Fludarabine 50mg d-5, -4, -3, Cyclophosphamide 600mg d-5, -4, 500mg d-3 as pretreatment. On October 21, 2020, autologous CD7 CAR-T cells were infused (50ml, 5*10^5^/KG). No lumbar puncture was performed after CAR-T cell therapy, and no neurological symptoms were observed.

The flow cytometry data for T cells after CAR-T can be found in **Figures S3-6**.

Figure S1: Flow cytometry of T cells after VDCLP regimen

**Figure S2** Flow cytometry plot of T cells after VDCLP schematization

**Figure S3** The flow cytometry data for T cells after CAR-T in d-2

**Figure S4** The flow cytometry data for T cells after CAR-T in 2-d14

**Figure S5** The flow cytometry data for T cells after CAR-T in 2-d36

**Figure S6** The flow cytometry data for T cells after CAR-T in 2-d110

**Table S1 Classification of complications and poor prognosis after the first CART infusion**

| Time | AE Type | CRS Grade | CRES Grade | Medication (Dose)/ Treatment |
| --- | --- | --- | --- | --- |
| d-3 | Fever（Tmax> 38℃）, | 0 | 0 | - |
| 2-d4 | Fever（Tmax> 38℃）, | 1 | 0 | anti-infection |
| 2-d7 | Fever（Tmax> 38℃）, Hypotension | 2 | 0 | anti-infection and fluid support |
| 2-d8 | Fever（Tmax> 38℃）  Blood pressure recovery | 1 | 0 | anti-infection and fluid support |
| 2-d9 | Fever（Tmax>＜37℃） | 0 | 0 | anti-infection and fluid support |

AE: Adverse Events, CRS: cytokine release syndrome, CRES: CART-related encephalopathy syndrome. The National Cancer Institute Common Terminology Criteria for Adverse Events (CTCAEv_5), and CRS was measured according to the ASTCT Consensus Grading, whereas CRES was measured using the CARTOX-10 criteria.
